# Evaluation of Diabetes Mellitus, Hyperlipidemia, and Thyroid Disease Comorbidities in Palmoplantar Pustulosis

**DOI:** 10.5152/eurasianjmed.2026.251026

**Published:** 2026-03-04

**Authors:** Ayşe Akbaş, Fadime Kılınç, Sertaç Şener, Yıldız Hayran

**Affiliations:** 1 Ankara City Hospital, Dermatology, Ankara, Türkiye; 2 Private Clinic, Ankara, Türkiye

**Keywords:** Diabetes mellitus, dyslipidemia, palmoplantar pustulosis, thyroid disease

## Abstract

**Background:**

Palmoplantar pustulosis (PPP) is a chronic, inflammatory pustular skin disease of unknown etiology. Genetic predisposition, smoking, focal infections, stress, and autoimmune diseases are implicated in its etiology.

In this study, the presence of thyroid disease, diabetes mellitus (DM), and dyslipidemia in PPP was investigated based on comorbidities in psoriasis.

**Methods:**

The records of 51 patients who were diagnosed with PPP both clinically and histopathologically and presented to the dermatology clinic were examined retrospectively. In these patients, fasting blood sugar, HbA1C, serum lipids (cholesterol, triglyceride, low-density lipoprotein (LDL), high-density lipoprotein (HDL)), thyroid-stimulating hormone (TSH), free thyroxine (ft4), free triiodothyronine (ft3), antithyroglobulin (antiTG), antithyroid peroxidase antibody (antiTPO) were recorded, and the presence of DM, dyslipidemia, and thyroid disease was investigated. Compared with the control group.

**Results:**

The average age of the patients was 47.9 (±12.1) years and the average disease duration was 5 (3-12) years. In the patients, hypertriglyceridemia was found in 29.4%, hypercholesterolemia in 45.1%, high LDL in 44%, low HDL in 14%, thyroid disease in 11.76%, and DM in 21.5%. When these values were compared with the control group, triglyceride values were significantly higher in the patients compared to the controls (*P *< .001).

**Conclusions:**

Based on the data of this study, as in psoriasis, it could be suggested that there is a possibility of DM, dyslipidemia, and thyroid disease comorbidity with PPP.

**Main Points**

- Palmoplantar pustulosis (PPP) is a chronic skin disease.
- It may be associated with some diseases such as hyperlipidemia, diabetes mellitus, and thyroid pathology.
- The presence of these diseases should be investigated and followed up in the diagnosis and treatment follow-up of PPP.

## Introduction

Palmoplantar pustulosis (PPP) is a chronic, recurrent, inflammatory skin disease characterized by sterile pustules and yellow-brown macules on an erythematous base on the palms of the hands and plantar surfaces of the feet.^1^ It affects 0.01%-0.05% of the population.^2^ Its pathogenesis is not fully known. It is more common in the age range of 45-70 and in females (58%-94%).^1^^,^^3^ While it was previously considered a variant of psoriasis by some authors, recent genetic studies have suggested that it is a separate entity.^4^^,^^5^ It adversely affects the quality of life of patients. Numerous triggering factors, such as genetic predisposition, smoking, focal infections, stress, autoimmune diseases, gluten enteropathy, and medications, are blamed in etiology.^5^

Today, as in psoriasis, the presence of numerous comorbidities such as thyroid pathologies, autoimmune and cardiovascular diseases, dyslipidemia, obesity, and metabolic syndrome has been investigated in PPP and a relationship with these diseases has been determined.^6^^-^^8^

Various studies have shown that PPP is accompanied by various comorbidities. The reason for the development of comorbidities in PPP is not fully understood. Various proinflammatory cytokines may affect lipid metabolism in this disease. Increased levels of IL-8 and IL-17A in PPP may indicate the role of immunological mechanisms.^1^

Various inflammatory mediators in PPP also affect insulin resistance.^9^^,^^10^ The aim of this study is to investigate the accompanying pathologies such as thyroid disease, diabetes mellitus, and dyslipidemia in the presence of PPP using a control group.

## Material and Methods

Prior to the study, approval was obtained from the local ethics committee (Decision number 176, dated July 11, 2018). The study was carried out in accordance with good clinical practice and the Declaration of Helsinki. Patient files from the dermatology department of Ankara City Hospital Dermatology Clinic between January 2019 and December 2023 were scanned. Patients' consent was obtained regarding the use of their data. This retrospective study is a single center case-control study.

In the study, 51 patients and 51 control files were examined.

Patients who applied to the dermatology clinic, received a clinical diagnosis of PPP in accordance with Navarini’s criteria, and whose diagnosis was also confirmed histopathologically, were included. Navarini’s criteria; PPP has primary, persistent (>3 months), sterile, macroscopically visible pustules on palms and/or soles and can occur with or without pustular psoriasis.^11^ Those using medications that could cause PPP were not included in the study. Patients whose diagnosis was not confirmed by biopsy were excluded from the study. From these patients’ files, data on age, gender, age of disease onset, disease duration, history of accompanying diseases, fasting blood glucose (FBG), HbA1c (if any), serum lipids (total cholesterol (TC), triglyceride (TG), low-density lipoprotein (LDL), high-density lipoprotein (HDL), thyroid function tests, and antibody levels; thyroid-stimulating hormone (TSH), free thyroxine (ft4), free triiodothyronine (ft3), antithyroglobulin (antiTG), antithyroid peroxidase antibody (antiTPO) were recorded. After the patient group was assembled, a healthy individual of similar age and gender was included in the control group for each participant in the patient group. Control patients were selected from patient files who were over 18 years of age, had no systemic disease, were not using systemic medication, and presented with complaints such as nevus, tinea infection, and verruca. Detailed family history of patients, the focus of focal infection, smoking use, and PPP severity scoring data could not be recorded as there was not enough data in patient records. Pregnant individuals, those with systemic diseases and neoplastic diseases, and those who have received systemic and topical drug treatment in the last 3 months were not included in the study. Patients were grouped by age range; 18-35 y, 36-55 y, 56 y and above. The place of residence was evaluated as the palmar, the plantar, and the palmoplantar. Normal reference laboratory values were taken as; FBG = 74-100 mg/dL, impaired glucose test FBG =100-125 mg/dL, HbA1c ≤ 5.7, thyroid function tests; ft3 = 1.8-4.6 ng/dL, ft4 = 0.9-1.7 ng/dL, TSH = 0.27-4.2 mU/L, antiTG = 0-115 IU/mL, antiTPO = 0-34 U/mL, TC = 50-100 mg/dL, TG = 0-200 mg/dL, HDL = 35-55 mg/dL, LDL = 0-130 mg/dL. Diabetes diagnosis was accepted as present if HbA1c ≥ 6.5 or FBG ≥ 126, and impaired glucose tolerance was accepted as present if FBG = 100-125 or HbA1c between 5.7 and 6.4. FBG**, **TC, TG, LDL, and HDL tests were performed using the spectrophotometric method on a Siemens Atellica CH Analyser. Thyroid function tests, anti-TG, and anti-TPO tests were performed using the electrochemiluminescence immunoassay method on a Siemens Atellica IM Analyser. HbA1c was performed using the capillary electrophoresis method on a Sebia Capillary 3 device. The recorded values were compared between the patient and control groups.

### Statistical Analysis

The data were analyzed using SPSS/IBM for Windows 21.0 (Chicago, IL, USA). Descriptive statistics such as percentage, mean, median, standard deviation, and interquartile range (IQR) were used to describe the sample. The assumption of normal distribution was examined with the Shapiro–Wilk test. The Student's *t*-test and ANOVA test were used in the cases where parametric test assumptions were provided, and the Mann–Whitney *U* and Kruskal–Wallis tests were used where these assumptions were not met. Spearman or Pearson tests were used to evaluate the correlation between 2 numerical values. Categorical data were analyzed with the chi-square significance test or Fisher's Exact test. A 95% significance level (*α *= 0.05 error margin) was used in the analyses to determine differences.

## Results

### Characteristics of Palmoplantar Pustulosis Patients

The study included 51 patients and 51 controls.

For patients = Average age 47.9 (SD = 12.1) years, female/male ratio= 1.55, male/female ratio = 0.65. Patients were divided into age groups. 19.6% were between 18 and 35, 58.5% were between 36 and 55, and 21% were over 55.

For the control group = Average age 48.8 (SD = 12.3) years, female/male ratio = 1.68, male/female ratio = 0.59.

Both groups were similar in terms of age and male/female ratio (*P* = .84, *P* = .70).

The incidence of low HDL was 66.7% in males and 33.3% in females. Low HDL was more common in males (*P* = .024). Other comorbidities were similar in males and females (all *P* values >.05).

The onset age for females was 44.1 (SD = 12.7) years, and for males, it was 50.7 (SD = 10.5) years. (*P* = .068). Disease durations were similar in males and females (*P* = .28). No relationship was found between age and disease duration (*P* = .59).

The presence of DM could not be detected in the 18-35 y age group. It was 11.7% in the 36-55 y age group and 37% in the group aged 56 y and over. The presence of DM was increasing with age (*P* = .003).

The presence of hypercholesterolemia was 14.3% in the 18-35 y age group, 42.6% in the 36-55 y age group, and 70.4% in the group aged 56 y and over. The presence of hypercholesterolemia was increasing with age (*P* = .002).

The incidence of high LDL was 23.1% in the 18-35 y age group, 34.4% in the 36-55 y age group, and 66.7% in the group aged 56 y and over. The presence of high LDL was increasing with age (*P* = .006). Other comorbidities were similar in all 3 age groups (all *P* values >.05).

No relationship was detected between the place of residence and the duration of the disease, among age groups, and among comorbidities. (*P* = .19, *P* = .87, *P* > .05).

No relationship was detected between lesion localizations and comorbidities (all *P* values >.05).

The average FBG was 103.8 (SD = 33.5) mg/dL in patients and 97.7 (SD = 34.1) mg/dL in the control group. Despite the high FBG in patients, it did not reach statistical significance (*P* = .081).

The median HbA1c was 7.1 (SD = 1.6) in patients and 6.1 (SD = 2) in the control group. HbA1c was significantly higher in patients (*P* = .010). 21.56% of patients and 14% of controls had DM. Patients and controls were similar in terms of the presence of diabetes (*P* = .45).

The average TCwas 199.6 mg/dL (SD = 45.9) in patients and 200.7 mg/dL (SD = 38.5) in the control group. Patients and controls were similar in terms of cholesterol (*P* = .71). Hypercholesterolemia (cholesterol >200 mg/dL) was present in 45.1% of patients and 47.1% of controls, and these rates were similar between the patient and control groups (*P* = .84)

The median TG was 145 mg/dL (IQR = 123-212) in patients and 115 mg/dL (IQR = 86-144) in the control group. TG was significantly higher in patients (*P* = .001). High TG (TG>200) was present in 29.4% of patients and 11.8% of the control group. High TG was significantly more common in patients (*P* = .028).

The average LDL was 125.2 mg/dL (SD = 38.1) in patients and 123.1 mg/dL (SD = 34.4) in the control group. Patients and controls were similar in terms of LDL (*P* = .88). High LDL (LDL>130) was present in 44% of patients and 40.8% of the control group. The patient and control groups were similar in terms of high LDL (*P* = .75).

The average HDL in patients was 47.5 (SD = 12.7) mg/dL and in the control group 49.7 (SD = 14.5) mg/dL. Patients and controls were similar in terms of cholesterol values (*P* = .31). Low HDL (HDL<35 mg/dL) was present in 14% of patients and 12.2% of the control group. The patient and control groups were similar in terms of low HDL (*P* = .81).

The average ft3 was 3.07 (SD = 0.48) ng/L in patients and 3.37 (SD = 0.52) ng/L in the control group. Ft3 values were significantly lower in patients (*P* = .009), but only one control group patient had ft3 levels outside normal limits.

The median ft4 was 1.19 (IQR = 1.08-1.31) ng/L in patients and 1.14 (IQR = 1.05-1.20) ng/L in the control group. Ft4 was significantly higher in patients (*P* = .034), but high ft4 was seen in 4 participants (2 patients, 2 controls). Low ft4 was similarly seen in 2 patients and 2 controls.

The median TSH was 1.93 (IQR = 1.42-3.33) mU/L in patients and 1.99 (IQR = 1.15-2.46) mU/L in the control group. Patients and controls were similar in terms of TSH (*P* = .21). High TSH (TSH>4.2 mU/L) was seen in 4 patients and 2 controls. The patient and control groups were similar in terms of high TSH (*P* = .39).

High anti-TG was present in 1 patient and 1 control. High anti-TPO was present in 1 patient and 1 control. Comparison of laboratory values with the control group was shown in Table 1. A history from patients revealed that a total of 6 patients had thyroid disease, 2 of whom were hypothyroid. Comparison of the presence of accompanying diseases was shown in Table 2.

## Discussion

PPP is a rare, inflammatory disease that can last for years with remissions and relapses. The chronic nature of the disease and the absence of a definitive cure negatively affect the quality of life in patients.^4^Various research has been conducted to date regarding the etiology of the disease, and many theories have been proposed, but no definitive conclusion has been reached.^1^^,^^5^

Some data obtained in this study show similarity with some literature when compared and differences in others.^1^^,^^12^^,^^13^ This difference is significantly influenced by the structure and number of the population.

PPP is generally a middle-aged disease. Many studies, including a very extensive study by Kim et al involving 37 999 PPP patients, found the average age to be between 38 and 53.^2^^,^^7^^-^^25^ In this study as well, the onset age was 47, similar to other studies. In this study, unlike other studies, it was found that the disease started later in men.

In all studies conducted, even though the populations differ, it is seen that PPP appears more frequently in females. This rate has been found to range from 51.7% to 88%in various studies.^8^^,^^13^^-^^18^^,^^23^^,^^24^^,^^26^^-^^28^ This may be due to females being more prone to autoimmune diseases.

In the extensive prevalence study conducted by Kubota et al in Japan, the male/female ratio was found to be 0.53. Similar ratios have been found in other studies (0.28-0.45).^11^^,^^29^ it was also found the male/female ratio to be a little higher at 0.65. A comparison of some demographic characteristics related to patients in various studies is shown in Table 3.

In the studies, the duration of the disease varies between 3.5-15 years.^7^^,^^12^^,^^13^^,^^23^^,^^24^^,^^26^ It was found that this rate as 5 years in the study. Females and males were similar in terms of disease durations. However, it was found that the disease started earlier in females.

In various studies, PPP has been evaluated in terms of involvement sites. Involvement of hands and feet has been found at different rates; these rates have been identified as 47%-73% for involvement of both hands and feet, 11.5%-61% for palms only, and 13%-36.17% for plantar only, indicating more common involvement of both hands and feet.^8^^,^^12^^,^^24^^-^^27^ The rates in this study are also shown in the Table 3.

Various meta-analysis studies have shown that PPP is accompanied by various comorbidities (Table 4). The reason for the development of comorbidity in PPP, like psoriasis, is not fully understood. Various pro-inflammatory cytokines may initiate the development of comorbidity by initiating lipid metabolism and immune cell traffic in this disease.^9^Increased levels of IL 8 and IL 17 A in PPP may indicate the role of immunological mechanisms.^1^

Various inflammatory mediators in PPP also affect insulin resistance, and severe involvement can facilitate the development of comorbidities. Some studies have suggested a relationship between PPP and type 2 DM.^23^^,^^28^ In these studies, it has been seen that diabetes accompanies PPP at various rates (7.7%-25%).^2^^,^^13^^,^^15^^,^^19^^-^^21^^,^^29^ In their studies conducted in the country, Ataş et al did not find a significant difference in FBG, insulin, and HOMA values in patients with PPP.^7^ The proportion of patients who had previously been diagnosed with DM in this study was found to be similar to some studies (patient group 21.5%, control group 14%).^20^^,^^28^ Although these rates were not statistically significant in this study, the fact that FBG and HbA1c values were higher in patients suggests that diabetes is more severe in patients.

In chronic inflammatory diseases, the relationship between dermatological diseases and dyslipidemia has been investigated due to the release of proinflammatory cytokines, and it has been found that dyslipidemia increases in these diseases.^10^Perhaps the most important element in the definition of dyslipidemia is low HDL. Recent studies have reported that HDL is not only responsible for lipid transfer, but also has a primary protective effect against atherosclerosis and plays an anti-inflammatory role.^30^

In this study, low HDL was 66.7% in males and 33.3% in females. Low HDL was more common in males. However, there was no statistical difference between them and the control group.

In this study, the rate of dyslipidemia was found to be quite high (45%-47%) in both PPP and control patients. This shows that dyslipidemia is common in Turkish society.

In various studies, dyslipidemia rates in PPP have been seen up to 49%.^2^^,^^12^^,^^15^^,^^19^^-^^21^ In these studies, data were evaluated under the heading of dyslipidemia. It was evaluated the data causing dyslipidemia, such as TC, HDL, LDL, TG, separately and saw that especially high TG was more accompanied and statistically significant. The significant elevation of TG values in this study compared to control groups, similar to other studies, clearly shows that there is dyslipidemia in patients.^23^^,^^28^ Hypertriglyceridemia may pave the way for PPP. Therefore, some authors suggest looking at the lipid profile as a baseline in PPP patients.^2^^,^^10^^,^^13^^,^^17^^,^^18^ Dyslipidemia was also detected in this study.

In their meta-analysis of 233 969 patients, Gleeson et al found diabetes mellitus in 12.6% and hyperlipidemia in 28.3% of patients with PPP, indicating that metabolic syndrome is more common in this disease.^31^

Kubota et al have suggested that the increases in dyslipidemia and diabetes in PPP may also be due to the side effects of the systemic treatments given.^2^However, in this study, patients were diagnosed with PPP for the first time, so there is no issue of medication use and side effects. Therefore, the developed dyslipidemia cannot be attributed to these factors.

In the general population, especially in females, the rate of thyroid disease is high.^6^^,^^23^ These rates are 2% for Hashimoto's, 1%-7% for overt hypothyroidism, and 12%-18% for subclinical hypothyroidism.^22^ Some researchers have found a relationship between PPP and thyroid diseases in their studies.^3^^,^^6^^,^^23^^,^^28^ In many studies, it has been shown that thyroid functions are impaired in PPP and there are autoantibodies related to the thyroid. These values can reach rates of 2.5%-25% accompanying hyperthyroidism or hypothyroidism.^6^^-^^8^^,^^10^^,^^12^^,^^13^^,^^15^^,^^23^^,^^27^ Although the exact cause of this abnormality is not known, it has been suggested that it binds to nuclear thyroid receptors involved in the regulation of skin keratin genes with thyroid hormones.^6^ A decrease in ft4 and an increase in anti-TPO increase the risk of developing hypothyroidism, Hashimoto's autoimmune thyroiditis.^23^ In this study, the frequency of patients who had previously been diagnosed with thyroid disease was consistent with the literature (11.7%, n = 6). There was no thyroid pathology in the control group. As a result of the screenings, abnormalities in thyroid functions were detected in an additional 6 patients. Screening thyroid function tests in patients with PPP can help with the early diagnosis of possible accompanying thyroid diseases.

### Limitation of Study

The limitations of the study include the fact that it is a single-center study; the number of patients is not large. Since this is a retrospective study, data on various factors thought to play a role in the etiology of PPP (family history, smoking history, focal infection focus, diet, obesity) could not be obtained from patient files.

In this study, it was observed that dyslipidemia, especially high triglycerides, may accompany PPP patients, and although the presence of DM is not significantly high compared to the control group, DM can be more severe. These patients should particularly be investigated for hypertriglyceridemia and thyroid disorders. The findings that was obtained in the study may pave the way for future research and treatment regimens.

## Figures and Tables

**Table 2.** Comparison of Presence of Accompanying Diseases

	**PPP**	**Control**	***P***
Number of patients	51	51	
Gender, m/f	0.65	0.59	.84
Presence of thyroid disease, %	11.76	0	–
Dyslipidemia, % High total cholesterol High triglyceride High LDL Low HDL	45.1 29.4 44 14	47.1 11.8 40 12.2	.84 .028 .75 .81
Diabetes mellitus	21.5	14	.45

**Table 3.** Comparison of Demographic Features of the Study with Some Studies in the Literature [7,8,12,16,20,23,24,25,27]

	**The Study 2024**	**Noe 2022 [25]**	**Sarıkaya Solak 2022 [24**]	**Olazagasti2017 [21]**	**Ataş2017 [7]**	**Tratter2016 [20]**	**Brunasso 2013 [12]**	**Adışen2009 [16]**	**Garcia 2003 [23]**	**Eriksson 1998 [8**]	**Enfors 1971 [27]**
Number of patients	51	197	263	215	33	102	39	52	17	59	248
F/M, (n)	20/31	52/145	98/165	36/179	2/31	15/87	13/26	37/63	4/13	7/52	22/78
Age of onset ( years)	47.9	53	42.38	45.3	40	42	48	40.2	40	42	20-60
Duration of disease years	5	3	4.6-5.59	6.5	4	5.8	6	3.8	3.5	1-56	2-10
Palmar involvement %	29.4	80	10.6	13	–	11.4	15.4	–	–	8	9 (6%)
Plantar involvement%	13.7	76	22.0	27	–	32.9	17.9	–	–	21	17(14%)
Palmar + plantar involvement %	56.9	59.9	60.1	60	–	55.7	66.7	73	–	69	74(61%)

**Table 4.** Comparison of Comorbidities Identified in the Study with Some Studies in the Literature [2,6,7,8,12,15,19,20,21,23,28]

	**The Study** **2024**	**Hiraiwa 2018 [** **19** **]**	**Ataş** **2017 [** **7** **]**	**Olagasti** **2017 [** **21** **]**	**Trattner** **2017 [** **20** **]**	**Kubata** **2015 [** **2** **]**	**Becher** **2014 [** **15** **]**		**Brunasso** **2013 [** **12** **]**	**Hagforsen** **2005 [** **28** **]**	**Garcia** **2003 [** **23** **]**	**Eriksson** **1998 [** **8** **]**	**Rosen** **1988 [** **6** **]**
Number of patients	51	136	33	215	102	13622	73	39		60	17	59	45
Total thyroid disease %, n	11.76 (n = 6)	4.4 (n = 6)	21 (n = 7)	8 (n = 18)	16	–	–	8 (n = 3)		11.6 (n = 7)	25 (n = 4)	13.56 (n = 8)	16 (n = 8)
DM type 2, %, n (7.7-18.6)	21.5	10.3 (n = 14)	–	10 (n = 21)	19	9.3	12.3 (n = 9)	–		25 (n = 15)	–	6.77 % n = 4)	–
Dyslipidemia, %, n (2-49)	45	4.4 (n = 6)	–	10 (n = 21)	38	18.3	49.3 (n = 36)	10.2 (n = 4)		–	–	–	–

**Table:** Table 1.Comprasion of laboratory values

	**PPP**	**Control**	***P***
Mean fasting blood sugar (SD)	103.8 (33.5)	97.7 (34.1)	.081
Mean HbA1c (SD)	7.1 (1.6)	6.1 (2)	.010
Mean ft3 (SD)	3.07 (0.48)	3.37 (0.52)	.009
Median ft4 (IQR)	1.19 (1.08-1.31)	1.14 (1.05-1.20)	.034
Median TSH (IQR)	1.93 (1.42-3.33)	1.99 (1.15-2.46)	.21
Mean total cholesterol (SD)	199.6 (45.9)	200.7 (38.5)	.71
Median triglyceride (IQR)	145 (123-212)	115 (86-144)	.001
Mean LDL (SD)	125.2 (38.1)	123.1 (34.4)	.88
Mean HDL (SD)	47.5 (12.7)	49.7 (14.5)	.31

IQR, interquartile range; SD, standard deviation.

## Data Availability

The data that support the findings of this study are available on request from the corresponding author.
